# Evolution of smoking rates among immigrants in France in the context of comprehensive tobacco control measures, and a decrease in the overall prevalence

**DOI:** 10.1186/s12889-023-15339-x

**Published:** 2023-03-15

**Authors:** Sarah Mahdjoub, Mégane Héron, Ramchandar Gomajee, Simon Ducarroz, Maria Melchior, Fabienne El-Khoury Lesueur

**Affiliations:** Sorbonne Université, INSERM UMR_S 1136, Institut Pierre Louis d’Épidémiologie Et de Santé Publique IPLESP, 27 Rue Chaligny, 75012 Paris, France

**Keywords:** Smoking, Smoking cessation, Geographic origin, Tobacco control

## Abstract

**Background:**

The evolution of smoking rates according to migrant status has not been examined in France, despite a recent reduction in overall smoking rates.

**Methods:**

DePICT is a two waves (2016: *n* = 4356; 2017: *n* = 4114) nationwide telephone survey, representative of the French adult population. We compared smoking-related behaviors before and after implementation of tobacco-control measures (2017), according to the geographical region of birth.

**Results:**

Compared to 2016, individuals originating from Africa or the Middle East had a slightly higher smoking prevalence in 2017 (34.7% vs 31.3%), despite a higher intention to quit or attempt in the preceding year (adjusted OR(ORa) = 2.72[1.90; 3.90]) compared to non-immigrants. They were also less likely to experience an unsuccessful quit attempt (ORa = 1.76[1.18; 2.62]).

**Conclusion:**

Tobacco-control measures could have widened smoking inequalities related to migrant status. The evolution of smoking-related behaviors among immigrants should be examined when studying the long-term effects of such policies.

## Background

Smoking prevalence has been declining over time in many Western countries, but it remains a leading cause of mortality and morbidity [1, 2]. This decline has been greatest among individuals with a high socio-economic position, making tobacco a major contributor to health inequalities [3, 4].

Some tobacco control policies and interventions are reported to be less effective among socially-disadvantaged individuals, which might contribute to the widening of inequalities with regard to smoking [5]. Therefore, the effect of tobacco control measures on equity should be systematically examined.

France has one of the highest smoking prevalence rates in the Western world [6]. After decades of stagnation at high smoking rates (around 30%), the country amplified tobacco control policies and introduced comprehensive measures in 2016. These measured consisted of the implementation of plain tobacco packaging, an increase in graphic health warnings on tobacco products, massive public health campaigns encouraging smoking cessation, and a planned increase in tobacco price [7]. These measures were followed by an unprecedented decrease in smoking rates among adults: in 2 years, there were 1.6 million fewer smokers among the French adult population (prevalence of regular smoking rates dropped from 29.4% in 2016 to 25.4% in 2018) [8, 9].

Auspiciously, these policies did not seem to widen socio-economic inequalities in this area. Until the COVID-19 epidemic, the decrease in smoking rates was comparable in individuals with low and high socioeconomic status, as defined by educational level [9, 10].

However, there is mounting evidence that marginalized social status due to an immigrant background, could drive health inequalities independently of education and income, especially due to marginalization and interpersonal and structural discrimination [11, 12].

Being an immigrant or having an immigrant background are now considered social determinants of health [13]. Immigrants and their offspring are often disadvantaged health-wise, compared to the general population. They are more likely to experience mental health problems, and steeper rates of health decline in older age [14, 15]. Several theories such as the acculturative stress – that is stress due to living in a foreign culture – and the cumulative disadvantage theory (migrants suffer from the negative effects of having a relatively low socioeconomic position throughout their life course) have been advanced to explain these differences [16, 17]. Further, migrants usually have low or inadequate health literacy compared to the general population [18].

In France, immigrants born in Africa and the Middle East make up the majority of the immigrant population, [19] and are reported to have worse health compared to individuals born in France [20] despite significantly lower smoking rates [21]. Therefore public health campaigns and tobacco control policies, as other preventive interventions, could have distinct impacts according to immigrant status due to different cultural backgrounds and social norms [22]. Understanding the impact of specific tobacco control measures on health inequalities is therefore important for developing and evaluating population-level public health policy interventions.

In this study, we investigated tobacco-related behaviors in France before and after the implementation of specific tobacco control measures, according to immigrant status as determined by the geographical region of birth.

## Methods

We conducted DePICT (Description des Perceptions, Images, et Comportements liés au Tabagisme), a nationwide telephone survey of residents of mainland France that took place in two waves one year apart: between the end of August and mid-November in 2016 and 2017. Therefore, the first wave took place before the implementation of several tobacco control measures such as plain packaging (January 1^st^ 2017), and smoking cessation media campaigns.

The target population consisted of all French speakers aged 18 to 64 years. Interviews were conducted via landline or mobile telephones by trained interviewers working for a polling institute located in the south of Paris. Randomly generated telephone lists were used to call participants up to 30 times using a computer-assisted telephone interviewing (CATI) system.

In households reached by landline, one participant was randomly selected by the CATI system (Kish method) [23].

### Ethical approval and informed consent

DePICT was approved by the ethical review committee of the French National Institute of Health and Medical Research (INSERM, CEEI-IRB 00,003,888). All procedures performed in studies involving human participants were in accordance with the ethical standards of the institutional research committee and with the 1964 Helsinki declaration and its later amendments or comparable ethical standard.

Informed consent was obtained from all individual participants included in the study.

### Measures

#### Smoking status, intentions to quit, and quit attempts in the preceding year

Participants were asked about their lifetime tobacco use and their current smoking status. Current smokers were asked about the daily number of cigarettes smoked, and whether they wished or tried to quit in the preceding 12 months (Y/N). Former smokers were asked about time since the last smoking cessation.

#### Geographical region of birth

Participants not born in France were asked about their geographical region of birth. We also asked participants about their parent’s geographic region of birth. We then classified individuals in four categories depending on whether they or their parents were born in: a) France (non-immigrant or direct descendant of immigrants), b) another European country (including Eastern Europe), c) an African or a Middle Eastern country, or d) another region. This categorization was motivated by previous research we conducted, where we showed that first and second-generation immigrants to France from Africa and/or the Middle East have different smoking patterns individuals born elsewhere [24, 25]. It was also motivated by the low number of smoking individuals from these “minority” groups in our study. Due to French regulations, [26] we were unable to ask more direct questions about perceived ethnicity, ethnic origin, or the country of birth. Due to their small effect size, first and second generation immigrants were grouped together.

### Socio-demographic characteristics and other covariables

We collected data on sociodemographic characteristics which have previously been linked to smoking: sex, age, educational level, and household situation [8]. Further, we also collected self-reported data on ever cannabis use and whether a participant lives with a smoker.

### Statistical analyses

To test the association between participants’ immigrant status and smoking cessation we proceeded as follows. For each study wave, data were weighted based on the probability of being selected through the Kish method (the ratio of the number of eligible individuals to the number of telephone lines in a household), [23] and to match the structure of the French population in 2016 for sex, age, education, region of residency and smoking experimentation rates, using data from the National Institute of Statistics and Economic Studies (INSEE) and the National Health Survey [27, 28]. We used the SAS raking macro to estimate a weight value to each participant, such that the weighted distribution of the overall sample is comparable to that with the listed variables in the 2016 French population [29].

In weighted descriptive analyses, we estimated smoking rates according to the study wave, and the geographic region of birth.

We also carried out two distinct multivariable regression models, to examine the adjusted association between the geographic region of birth and two different outcomes among smokers or former smokers.

The first model was used to determine the adjusted association (ORa) between the geographic region of birth and the intention or attempt to quit in the preceding year (Yes/No) among smokers, adjusting for covariates, which included characteristics previously linked to smoking, which were significantly associated with the study outcome in bivariate analyses.

The second multivariable logistic regression model was limited to smokers who intended to or attempted to quit smoking in the preceding year, and former smokers who quit in the preceding year. We therefore examined factors associated with an unsuccessful quit attempt in the preceding year (***Yes***: smokers who intended to or attempted to quit in the preceding year vs. ***No***: Former smokers in the preceding year).

All statistical analyses were conducted using SAS version 9.4 (SAS Institute Inc), statistical significance was set to 0.05.

## Results

### Smoking rates

**Fig. 1:**
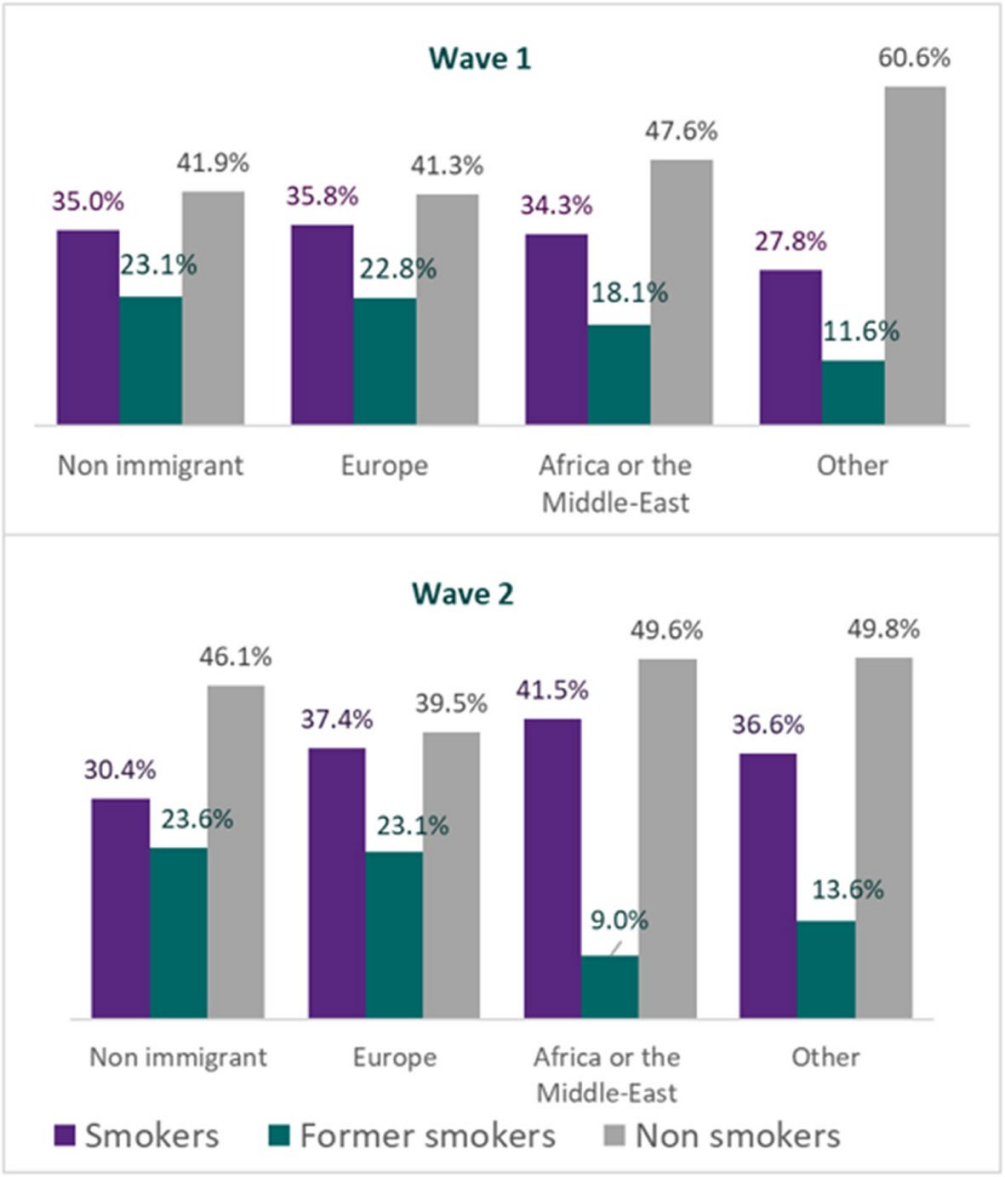
Smoking status (weighted prevalence (%)) in 2016 and 2017 according to participants or their parents’ geographical region of birth (total *n* = 8470; first study wave (2016): *n* = 4356, second study wave (2017): *n* = 4114)

We recruited a total of 8470 participants (2016: *n* = 4356; 2017: *n* = 4144), with an unweighted mean age of 44 [sd = 13; weighted mean = 42 (sd = 13)]. More than half of the participants were women (53%, weighted percent = 51%), and people with no high school diploma were under-represented in the original sample (unweighted percent: 31%; weighted percent: 47%). Overall, the percentage of smokers significantly decreased between the first and second wave (weighted percent, 2016: 34.7%; 2017: 32.3%; *p* = 0.022). However, among individuals born in sub-Saharan Africa, North Africa or in the Middle East (AfrME-origin) the percentage of smokers significantly increased by 6.2% between the two study waves (37.3% vs 41.5%; *p* = 0.023). There were (non-significantly) more former smokers in the general population in the second study wave compared to the first (weighted %: 23.2% vs. 22.6%; *p* = 0.7), while the proportion of former smokers among participants of AfrME-origin significantly decreased (18.1% vs 9.0%; *p* < 0.001) (Fig. 1).

### Smoking quit attempts in the last year

Smokers’ characteristics according to their intention or attempt to quit in the preceding year, are presented in Table 1. Less than half of smokers in our study (*n* = 2269) were women (weighted percent: 43.6%). The average age of smokers was 39 years (sd = 14.3), and AfrME-origin individuals constituted 12.7% (weighted percent) of the smokers’ population.

**Table 1:** Characteristics of smokers participating in the DEPICT study (weighted percent), according to their intention or attempt to quit smoking in the preceding year” (*n* = 2261)

	**Total population *n* = 2261**	**Quit attempt or desire to quit in the last year**		***p*-value (Chi-Square Test)**
		No (unweighted *n* = 535)	Yes (unweighted *n* = 1726)	
**Region of birth**				< .0001
France	1758 (74.5%)	24.4%	75.6%	
Europe	207 (9.5%)	20.1%	79.9%	
Africa or the Middle-East	234 (12.7%)	11.5%	88.5%	
Other	70 (3.4%)	19.6%	80.4%	
**Study wave**				0.52
First (2017)	969 (46.8%)	22.7%	77.3%	
Second (2016)	1292 (53.2%)	21.7%	78.3%	
**Sex**				0.86
Men	1201 (56.4%)	22.3%	77.7%	
Women	1060 (43.6%)	22.1%	77.9%	
**Age**				0.07
< 30	525 (30.2%)	25.0%	75.0%	
≥ 30 et < 45	734 (34.6%)	21.0%	79.%	
≥ 45	1002 (35.2%)	21.1%	78.9%	
**Educational level**				0.001
No High school diploma (< Bac)	834 (55.0%)	21.0%	79.0%	
High School or two-year university degree	885 (32.0%)	21.4%	78.6%	
At least a three-year university degree	542 (13.0%)	29.6%	70.4%	
**Household situation**				0.08
Doesn’t live with a smoker	696 (27.7%)	23.9%	76.1%	
Lives alone	843 (39.6%)	23.1%	76.9%	
Lives with a smoker	722 (32.7%)	19.8%	80.2%	
**Number of cigarettes smoked /day**				< .0001
< 10	876 (35.1%)	29.0%	70.1%	
≥ 10	1291 (64.9%)	17.5%	82.5%	
Missing	94	40	54	
**Ever cannabis use**				0.37
No	1272 (55.8%)	21.6%	78.4%	
Yes	986 (44.2%)	23.02%	77.0%	
Missing	3	1	2	

Quit attempt or desire to quit was especially high among individuals from the AFR-ME group (88.5% vs 11.5%) compared to other groups (other European migrants: 79.9% vs 20.1).

The results of the multivariable analysis (Table 2) show that AfrME-origin smokers were more likely to report the intention or attempt to quit in the preceding year (ORa = 2.72 [1.90–3.90]) compared to non-immigrants or direct descendant of immigrants.

**Table 2:** Determinant of “quit attempt or desire to quit in the last year” (Yes vs No) among smokers in the DePICT study (*n* = 2164): results of the multivariable logistic regression model, OR; 95% CI

	**OR intention or attempt to quit in the preceding year (Yes vs No)**
**Region of origin (ref: France)**	
Europe	1.28 (0.92; 1.78)
Africa or the Middle-East	**2.72 (1.90; 3.90)**
Other	1.30 (0.77; 2.19)
**Study wave (ref: first)**	
Second (2017)	1.15 (0.95; 1.38)
**Sex (ref: men)**	
Women	1.08 (0.89; 1.31)
**Age (ref: < 30)**	
≥ 30 et < 45	1.17 (0.93; 1.48)
≥ 45	1.19 (0.93; 1.53)
**Educational level (ref: High School or two year university degree)**	
No High school diploma (< Bac)	0.81 (0.65; 1.01)
At least a three year university degree	0.69 (0.51; 0.92)
**Living situation (ref: doesn’t live with a smoker)**	
Lives alone	0.99 (0.79; 1.25)
Lives with a smoker	**1.29 (1.01; 1.64)**
**Number of cigarettes smoked (ref: < 10)**	
≥ 10	**1.96 (1.61; 2.39)**
**Ever cannabis use (ref: no)**	
Yes	0.96 (0.78; 1.17)

*ref* reference category; the *p*-value is strictly less than 0.05 for ORs (95%CI) in bold characters (confidence interval does not contain the value 1)

### Quit attempt in the preceding year

For this model, the sample consisted of participants who quit smoking in the preceding year (*n* = 370) and smokers who desired or attempted to quit in the preceding year (*n* = 1734).

The results of the multivariable analysis (Table 3) show that AfrMe-origin individuals were more likely to have had an unsuccessful smoking attempt in the preceding year compared to participants with France as the region of birth (ORa = 1.76 [1.18—2.62]).

**Table 3:** Determinant of smoking cessation in the preceding year: results of the multivariable logistic regression model, OR; 95% CI. Depict study, 2016 and 2017, *n* = 2 104

	**Smokers who desired or attempted to quit in the last year (vs ex-smokers who stopped in the last year)**
**Region of origin (ref: France)**	
European	1.29 (0.85; 1.96)
African or the Middle-East	**1.76 (1.18; 2.62)**
Other	1.76 (0.81; 3.83)
**Study wave (ref: first)**	
Second (2017)	0.89 (0.70; 1.12)
**Sex (ref: men)**	
Women	0.88 (0.70; 1.11)
**Age (ref: < 30)**	
≥ 30 and" < 45	***0.67 (0.50; 0.91)***
≥ 45	0.75 (0.55; 1.03)
**Educational level (ref: High School or two year university degree)**	
No High school diploma (< Bac)	**2.07 (1.59; 2.69)**
At least a three year university degree	0.80 (0.58; 1.10)
**Living situation (ref: Doesn’t live with a smoker)**	
Lives alone	**1.91 (1.47; 2.48)**
Lives with a smoker	**2.93 (2.16; 3.97)**

The *p*-value is strictly less than 0.05 for ORs (95%CI) in bold characters (confidence interval does not contain the value 1)

## Discussion

Our results, based on data from a two-wave nationally representative repeated cross-sectional study of 8 470 individuals in France in 2016 and 2017, show that despite an overall decrease in smoking rates after the intensification of tobacco control measures, smoking rates appear to have increased among individuals with an immigration background. In particular, individuals born in Africa or the Middle East, who comprise the largest part of immigrants in France, reported significantly higher levels of quit attempts, but an increased smoking prevalence.

There is considerable literature on the association between migrant status and unhealthy behaviors in high-income countries. Generally, migrant groups have lower levels of some healthy behaviors such as access to preventative health services (including cancer screening) and physical activity compared to the general population [30, 31]. Further, longer durations of residence are linked with the acquisition of unhealthy behaviors such as unhealthy diet and smoking among migrants [32, 33]. In France, pre-migration prevalence of smoking is generally lower among African migrants arriving in the country. However, this prevalence tends to increase with time, up to levels beyond those of the native-born for certain male migrant groups, while migrant women tend to have significantly lower smoking prevalence compared to the French female general population [34]. This increase in unhealthy behaviors with time among migrants is likely exacerbated by low socio-economic disadvantage, cumulative exposure to racism, and low health literacy [31, 35]. There is also evidence that some public health interventions, which improve overall population health, could lead to ‘intervention generated inequalities’ [36, 37]. However, there is very little data on effective interventions to improve immigrant health, especially from Europe, with experts calling for more data from natural experiments like changes in policy [38]. We advance this literature by describing how comprehensive tobacco control policies in France, which were successful in decreasing overall smoking rates, did not lower smoking rates among migrants and descendant of immigrants.

Tobacco control measures may have had comparable – if not better—effects on the desire to quit among immigrants. However, even if smokers born in Africa or the Middle East reported a higher desire and quit attempts, their success rates seem to be lower compared to the general population. Lower quit rates among immigrants could be explained by low access to smoking cessation services (general practitioners and tobacco cessation and addiction specialists), which is common among individuals with low socioeconomic status [39]. It could also be explained by a poorer mental health, and lower health literacy. Other mechanisms could also explain our results, such as a surge in illicit (and cheaper) cigarettes from African and middle eastern countries being sold on the streets. However, little data is available on this subject.

These findings could imply that the prevalence of smoking among some immigrants and descendants of immigrants in France increases with time. This is in accordance with other European studies which also found disparities in smoking rates according to migrant status and acculturation [40].

Our findings suggest that tobacco control strategies should provide specific measures to increase successful quit attempts rates among marginalized populations. Prevention and smoking cessation interventions tailored specifically to first and generation immigrants—such as neighborhood-based and/or culturally tailored programs—are needed.

The evaluation of public health interventions should also systematically include effects on migrants and other minority populations.

### Limitations

Our study is one of the first to examine the change in smoking rates among immigrants after the implementation of new tobacco control measures. However, some limitations need to be noted. First, selective non-response to our repeated survey could have resulted in selection bias, especially if smokers were less inclined to participate. It is possible that smokers were more reluctant to participate in the second wave compared to the first because of a perceived increase in the stigmatisation of smoking. Nevertheless, we did weigh study data to limit such bias. Second, as in most other epidemiological studies, we use self‐reported data on smoking, which may have resulted in under-estimating smoking rates. Further, language barrier could also be a limitation in this survey targeting solely the French-speaking population. Moreover, merging immigrants and descendant of immigrants due to small effect size is likely to conceal differential subgroups trends. We also did not stratify analysis by sex due to small effect size.

## Conclusions

Smoking rates appear to have increased among individuals with an immigration background in France, despite the intensification of tobacco control measures and a decrease in smoking rates among the general population. Our study provides evidence suggesting that the effect of tobacco control measures could have different effects depending on the smokers’ migrant status.

## Data Availability

Available upon request from corresponding author, at fabienne.khoury(at)inserm(.)fr.

## Abbreviations

DePICT: Description des Perceptions, Images, et Comportements liés au Tabagisme
CATI: Computer-assisted telephone interviewing
INSEE: French National Institute of Statistics and Economic Studies
ORa: Adjusted odds ratio
AfrME-origin: Individuals born in Africa or in the Middle East
Sd: Standard deviation
Ref: Reference category
